# Psychosocial and Neurocognitive Factors Associated With Hepatitis C – Implications for Future Health and Wellbeing

**DOI:** 10.3389/fpsyg.2018.02666

**Published:** 2019-01-09

**Authors:** David Pires Barreira, Rui Tato Marinho, Manuel Bicho, Renata Fialho, Silvia Raquel Soares Ouakinin

**Affiliations:** ^1^Clínica Universitária de Psiquiatria e Psicologia Médica, Faculdade de Medicina, Universidade de Lisboa, Serviço de Gastrenterologia e Hepatologia, Centro Hospitalar Lisboa Norte-Hospital de Santa Maria, Lisbon, Portugal; ^2^Faculdade de Medicina, Universidade de Lisboa, Serviço de Gastrenterologia e Hepatologia, Centro Hospitalar Lisboa Norte-Hospital de Santa Maria, Lisbon, Portugal; ^3^Laboratório de Genética, Faculdade de Medicina, Instituto de Saúde Ambiental, Universidade de Lisboa, Lisbon, Portugal; ^4^Assertive Outreach Team, Sussex Partnership NHS Foundation Trust, Brighton and Hove, United Kingdom; ^5^Clínica Universitária de Psiquiatria e Psicologia Médica, Faculdade de Medicina, Universidade de Lisboa, Lisbon, Portugal

**Keywords:** hepatitis C, neurocognition, adherence, wellbeing, psychosocial factors

## Abstract

**Background:** Hepatitis C virus (HCV) infection involves changes not only from the point of view of physical health, but also emotional, and social that have a significant impact on the quality of life of these patients. According to the literature review, it seems that there is an important association between psychosocial factors, in particular on a cognitive level and disease progression. The aim of this mini-review is to summarize recent literature looking at the associations between psychosocial and neurocognitive factors and HCV.

**Methods:** PubMed/Medline was systematically searched for psychosocial and neurocognitive factors associated with hepatitis C, treatment adherence, and patient wellbeing.

**Results:** Patients present with a range of extrahepatic symptoms including fatigue, anxiety, depression, and neurocognitive dysfunction. HCV’s impact on quality of life and wellbeing has serious clinical and social implications.

**Conclusion:** Hepatitis C and its management continue to have a profound impact on health and psychologic wellbeing. Considering the serious extrahepatic implications for individuals, it is imperative that healthcare professionals pay close attention to psychosocial and neurocognitive factors. The focus on combined clinical approaches could enhance understanding about the health and social impacts of hepatitis C along the life course.

## Introduction

The World Health Organization (WHO) estimates that about 3% of the world population (170 million people) is infected with the hepatitis C virus (HCV) (WHO, 1999). The disease’s potential for evolution toward chronicity, hepatic cirrhosis, and hepatocellular carcinoma makes this the main indicator for a liver transplant (Marinho and Barreira, 2013) before effective therapies arise. Treatment for HCV changed in the last years from an interferon and ribavirin-based therapy to being interferon-free. These are simpler oral regimens, shorter to administer with very high efficacy rates and better side effect profiles (Smith et al., 2015). However, as advances in the treatment of HCV occur, it is imperative to evaluate clinical outcomes, assessing and quantifying the efficacy and impact of these regimens.

The identification of neuropsychiatric and neurocognitive symptoms is important during and before treatment; however, they still have little recognition or impact in clinical evaluation. Further, these symptoms (depression, cognitive disorders) are eventually associated to direct HCV neurotoxicity (Adinolfi et al., 2015), negatively affecting the individual’s perceived Quality of Life (QoL), daily functioning, work, and productivity. These factors can lead to reduced involvement in medical care and life projects as well as increased morbidity and mortality, decreasing overall wellbeing, QoL, as well as treatment adherence (Younossi et al., 2007; Schaefer et al., 2012; Adinolfi et al., 2015; Monaco et al., 2015; Younossi and Henry, 2015; Chasser et al., 2017).

In a biopsychosocial model of the disease and the treatment, it is essential to understand HCV’s clinical and social impact on a broader life perspective and how individuals experience it. The basic principles of this model, which uses a holistic approach regarding illness, includes the biological, psychological, and social dimensions of the person’s life and the perception that the person suffers as a whole. The personality and the emotional reserves of the patient as well as the particular environmental conditions in which the person lives should be taken into account (Papadimitriou, 2017). On the other hand, positive psychology and focusing on wellbeing have inspired research into the aspect of the relation between emotion and health; namely, the relationship between subjective wellbeing and health outcomes (Okely and Gale, 2015). Wellbeing, in the context of a chronic condition such as HCV infection, must follow the WHO’s inclusive definition of health and states of wellbeing (Misselbrook, 2014). The literature explains this construct as a combination of three different dimensions, each capturing a different aspect: life evaluation (quality or goodness of ones’ life and overall life satisfaction), hedonic wellbeing, and eudemonic wellbeing. The hedonic dimension refers to the experience of positive and negative affect (e.g., sadness or happiness) and not to a unique positive affective valence, while the eudemonic dimension captures meaning and purpose in life (Steptoe et al., 2015). Thus, the aim of this article is to determine what is currently known about neurocognitive and psychosocial factors associated with HCV as well as to try to value disease adjustment and the implications for health and wellbeing.

## Methods

The aim of this mini-review is to summarize the more recent literature investigating the relationship between psychosocial and neurocognitive factors associated with HCV and their impact on patient wellbeing. We searched the Medline/PubMed databases. No time period limits were placed, and the key terms included “psychosocial,” “neurocognitive,” “hepatitis C,” and “wellbeing.” In addition to this selection, some research gathered outside this search was reported as evidence, if considered appropriate by all authors.

## Psychosocial Factors and Hepatitis C

The chronic nature and individual experience of the disease are conditioning factors on QoL, with clinical and social implications, due not only to the diagnostic but also to its evolution (Negro et al., 2015; Chasser et al., 2017; Iriana et al., 2017). Several studies have stated that chronic hepatitis C may lead to several complications anddespite the fact that most of the patients with HCV are asymptomatic, they consistently report a significant reduction in health-related QoL, when compared with the general population (Baune and Air, 2016; Adinolfi et al., 2017).

Psychosocial chronic stressors are well documented as determinants of poor mental and physical health, leading to an important burden in health systems, mortality, morbidity, and psychological wellbeing, predominantly in developed societies (Yarlott et al., 2017). The relationship between physical mental and social health as well as the uncertainties about treatment and HCV have been well documented, both in the past and in recent literature (Hong et al., 2011; Kleinman et al., 2012; Armstrong et al., 2016). Emotional distress and depressive disorders have been reported in untreated HCV patients, pointing to a possible role of the virus itself in their occurrence (Alavi et al., 2012; El Khoury et al., 2012). Furthermore, studies suggest that a deficiency of social relationships is a significant risk factor for broad-based morbidity and mortality as well as negative implications for health (Cacioppo and Cacioppo, 2014; Valtorta et al., 2016). Whether these disorders are due to the uncertainty of living with a chronic disease with potentially life-threatening complications or to other psychosocial factors, remains under discussion.

Another relevant factor is the stigma associated with HCV, which may increase anxiety levels and the fear of transmitting the virus. This fear may be the main cause for social isolation and decreased intimacy in relationships (Younossi et al., 2007; Armstrong et al., 2016). Stigma may be defined as a cluster of attitudes expressed by a dominant group, which sees other individuals’ behaviors as being socially unacceptable. The notion of stigma, related to shameful relationships, and deviations from what is considered to be the “norm,” has a long history in the context of infectious diseases, as in the case of HCV (Bogart et al., 2008). These norms, behaviors, and beliefs may lead to alienation of family and social relationships as well as (real or perceived) to discrimination at health settings or the workplace.

Stigmatization affects not only patients but also health care professionals who are not immune to stereotypes and judgments that may influence treatment. These issues can promote an increase in patients’ isolation, in therapeutic continuity, and a decrease in the search for medical help (Butt, 2008). Furthermore, several studies have suggested that stigma is associated with low treatment adherence (Kamaradova et al., 2016), including in HCV patients (Treloar et al., 2013). Implementing psychological strategies and intervention models (e.g., psychoeducation; information-motivation-behavioral skills) should be a major concern.

A recent systematic review has shown that non-adherence to the HCV treatment regimen is associated with virologic response failure (Lieveld et al., 2013), supporting that optimal adherence is needed to achieve treatment success in this area (Mathes et al., 2014). This also becomes a public health issue, since non-adherence favors the development and spread of resistant HCV mutations, highlighting adherence’s crucial role in treatment success (Weiss et al., 2009).

Investigations about medication adherence indicate the need to analyze interconnected factors related to the complexity of therapeutic regimes (treatment of HCV and comorbidities), relevance of side effects (nausea, pruritus, insomnia, diarrhea, and asthenia) (Feld et al., 2014), but also patient-related variables. Research points to a steady correlation between individual characteristics, illness behaviors, health literacy, and low adherence to treatment. Among sociodemographic and clinical factors, gender, depression, psychiatric diagnosis in general, illicit drug use, HIV co-infection, treatment regimen, and hemoglobin level were identified as being significantly and consistently associated with adherence/non-adherence. Age, race, unemployment, being unmarried, genotype, treatment experience, disease severity, and HCV-related costs were classified as inconsistent predictors of non-adherence (Lieveld et al., 2013; Mathes et al., 2014).

From a psychological point of view, factors such as information, personal beliefs, personality traits, social context and the way they interact toward health-promoting behaviors, allow a better understanding of adherence bases, helping to implement individualized adaptive strategies. Further, the general physical symptomatology related to HCV can vary from musculoskeletal discomfort to chronic fatigue (Armstrong et al., 2016). In a recent study, Boscarino et al. (2015) reported that poor physical health in patients with HCV was associated with demographic factors, including income, health insurance status, marital status, and also connected with stressful life events, social support or having a liver transplant. Despite the needed level of adherence (95% or higher), non-adherence should not be a factor of exclusion to initiate antiviral treatment, but rather a factor that induces a careful evaluation in the stage of pre-prescription, as well as the implementation of strategies designed to promote adherence in the context of a multi-disciplinary team. Thus, we argue that an integrated medical and psychological approach is fundamental, being associated with a higher adherence and to a better therapeutic response.

The following steps have proven to be crucial for the success of the treatment: to evaluate the patient’s preparation to start and adhere to the therapeutic regime, to choose a therapeutic regime that may improve adherence, and to select and to apply interventions during the therapeutic regime.

Hepatitis C virus diagnosis is reported by individuals as having a profound impact on their social functioning and wellbeing. Psychosocial factors (social support, coping mechanisms) not only interfere in the way the subject interprets and experiences symptoms, but they also influence the medical treatment and modify behaviors (Bielski and Chan, 1976).

## Neurocognition and Hepatitis C

The involvement of the central nervous system (CNS), found in recent studies, supports a pathogenic role for HCV in neuropsychiatric and neurocognitive disorders (Monaco et al., 2015). In a recent review, Yarlott et al. (2017) called this association between HCV, cognitive impairment, fatigue and depression the “Hepatitis C Virus Brain Syndrome.” This syndrome is probably generated by peripheral immune responses affecting the CNS, neuroinflammation associated with CNS HCV infection, as well as to negative life events and other psychogenic stressors (Yarlott et al., 2017).

Forton et al. (2001) showed evidence that HCV can cross the blood-brain barrier and replicate in the CNS, emerging through infected monocytes and infecting microglial cells. Using proton magnetic resonance spectroscopy in patients and controls, they found an increase in basal ganglia and white matter choline/creatine ratios in patients, suggesting an altered metabolism in patients with chronic HCV; these results were not explained by hepatic encephalopathy or history of drug use (Forton et al., 2001).

Neurocognitive impairment, one of the most common extrahepatic manifestations of HCV, can lead to subtle changes in processing speed, memory, attention, fatigue, and cognitive performance. Up to 50% of HCV-infected patients may develop clinical or subclinical manifestations of this dysfunction, and these cognitive deficits are independent of the stage of the liver disease (Lowry et al., 2010; Ferri et al., 2016; Iriana et al., 2017). Hilsabeck et al. (2002) found that HCV patients experience cognitive deficits, especially in attention, learning, psychomotor speed, and mental flexibility. This study shows that maintaining attention and concentration while performing accurately was the most difficult task for non-cirrhotic patients, with 50% taking an abnormally long time to complete the task, 28.9% making a significant number of omission errors, and about 20% of non-cirrhotic patients performing in the impaired range involving attention/concentration, visual scanning and tracking, psychomotor speed, and mental flexibility (Hilsabeck et al., 2002). The authors suggest that this cognitive impairment is similar to that reported in patients with a neurocognitive disorder associated to other chronic illness, such as HIV and AIDS-related dementia, and that these deficits affect QoL and functional capacity in patients (Hilsabeck et al., 2002).

Regarding cognitive and psychiatric dysfunctions, several studies were performed to assess the impact of HCV in patients, namely depression, anxiety, fatigue, attention, executive function, and visuospatial function deficits (Monaco et al., 2015; Thames et al., 2015). Cognitive impairments were previously thought to be associated with the development of hepatic encephalopathy (Gaeta et al., 2013). However, their presence was demonstrated in the absence of advanced liver disease as well as in the absence of HIV co-infection, depression, or substance abuse (Cherner et al., 2005; Kuhn et al., 2017; Yarlott et al., 2017). Despite the absence of those comorbidities, deficits in attention, concentration, psychomotor speed, and verbal fluency have been reported in HCV patients (Weissenborn et al., 2004; Clifford et al., 2009). Findings remain controversial and it is unclear if HCV eradication improves HCV-associated neurological compromise (Kuhn et al., 2017). Bladowska et al. (2013) in a follow-up study over 48 weeks after the cessation of treatment, found significant improvement in attention and working memory in patients who successfully cleared the virus. This finding suggests that over time, the benefits of HCV clearance can be more marked. However, in other research protocols sustained virologic response (SVR) was not related to any improvement in neurocognitive performance (Huckans et al., 2015; Lowry et al., 2016).

Therefore, the neurocognitive profile of these patients before treatment needs to be addressed due to the possible implications on the treatment course (e.g., non-adherence-like behavior due to cognitive abnormalities). Additionally, it is important to address other risk factors that can have a negative impact on cognition. The high rate of substance abuse and the prevalence of psychiatric disorders among HCV-infected patients are important factors associated with cognitive impairment that trigger poor adherence and treatment efficacy (Wiêdłocha et al., 2017).

Several studies have suggested that earlier treatment is associated with improvement of the QoL in chronic HCV patients (Marinho and Barreira, 2013; Boscarino et al., 2015; Lucaciu and Dumitrascu, 2015; Negro et al., 2015; Chasser et al., 2017; Iriana et al., 2017), highlighting the importance of HCV treatment. In order to optimize treatment outcomes, a pretreatment profile helps to identify risk factors that can be modifiable before and during treatment, such as treatment disruption, non-adherence, the risk of (re)infection and risk of emergence of depressive disorders (Basseri et al., 2010).

The pre-vs.-post treatment neurocognitive changes that may occur among SVR patients require further research to understand the impact of neuroanatomical and functional changes in HCV patients before, over the course of treatment, and after successful clearance of the virus.

## Discussion and Conclusion

This mini-review shows that patients with HCV often feel stigmatized and unsupported in their care, relationships, and work environments, while simultaneously coping with physical and psychological symptoms. This synthesis points to areas where greater education, compassion, and patient-centered healthcare could improve the experience of people living with HCV.

In a broader perspective, and trying to close the loop, neurocognitive impairment can be responsible for therapy non-adherence in several diseases (Rohde et al., 2017; Smith et al., 2017) and for losing internal resources to deal with a chronic disease (Boehmer et al., 2016). Adaptive behavior is therefore impaired, social networks are disrupted and patients’ deficits in instrumental daily life abilities are increased. These are factors known to be associated to a worse QoL (Vilhena et al., 2014), potential complications in disease evolution (Vere, 2009), and overall a worse prognosis. Whether, mediating variables in this process include individual psychological traits, negative or positive affective states, cognitive impairment, biological responses associated to more pathogenic viral stripes, or to the combination of multiple disease-factors, is yet an open debate. However, the relevance of those psychosocial variables in chronic diseases is nowadays unquestionable (Fava et al., 2016; Kemp, 2017; McLachlan and Gale, 2018). In what concerns wellbeing, all the described dimensions may be affected by psychosocial factors and they can be managed through an integrated intervention. Actions on preventing, identifying and treating hepatitis C should be a priority and need to be included in local health and wellbeing strategies in order to promote better health outcomes.

The association between neurocognitive functions, psychological wellbeing, and activities of daily living among patients with hepatitis C has had little attention. Understanding factors, which can impact physical functioning and psychological wellbeing, may have an important clinical relation to improving outcomes for patients with this disease.

Treatment of HCV requires education efforts of a wide base, with the goal of improving knowledge, and attitudes about this disease. These efforts must include patients and their families, policy decisions, health care professionals, and society as a whole. A thorough study of HCV-associated stigma, clinical, and social implications is essential for helping patients to cope with the disease.

Investigation supports that HCV experience and treatment adjustment may be facilitated through the use of theoretical informed psychological interventions. Considering the impact of HCV infection in patients’ mental health and wellbeing, before, and during treatment, an interdisciplinary approach should be followed and encouraged. Besides HCV-associated depression, more studies are needed to characterize the eventual neurocognitive side effects of the new interferon-free regimes and their impact on mood comorbidities.

## Author Contributions

All authors listed have made a substantial, direct and intellectual contribution to the work, and approved it for publication.

## Conflict of Interest Statement

The authors declare that the research was conducted in the absence of any commercial or financial relationships that could be construed as a potential conflict of interest.
